# Increased glomerular filtration rate and impaired contractile function of mesangial cells in TRPC6 knockout mice

**DOI:** 10.1038/s41598-017-04067-z

**Published:** 2017-06-23

**Authors:** Weizu Li, Yanfeng Ding, Crystal Smedley, Yanxia Wang, Sarika Chaudhari, Lutz Birnbaumer, Rong Ma

**Affiliations:** 1 Institute for Cardiovascular and Metabolic Disease, University of North Texas Health Science Center, Fort Worth, Texas 76107 USA; 2 Department of Pharmacology, Anhui Medical University, Hefei, Anhui 230032 P. R. China; 3 Transmembrane Signaling Group, National Institute of Environmental Health Sciences, National Institutes of Health, Research Triangle Park, North Carolina, 27709 USA

## Abstract

The present study was conducted to determine if TRPC6 regulates glomerular filtration rate (GFR) and the contractile function of glomerular mesangial cells (MCs). GFR was assessed in conscious TRPC6 wild type and knockout mice, and in anesthetized rats with and without *in vivo* knockdown of TRPC6 in kidneys. We found that GFR was significantly greater, and serum creatinine level was significantly lower in TRPC6 deficient mice. Consistently, local knockdown of TRPC6 in kidney using TRPC6 specific shRNA construct significantly attenuated Ang II-induced GFR decline in rats. Furthermore, Ang II-stimulated contraction and Ca^2+^ entry were significantly suppressed in primary MCs isolated from TRPC6 deficient mice, and the Ca^2+^ response could be rescued by re-introducing TRPC6. Moreover, inhibition of reverse mode of Na^+^-Ca^2+^ exchange by KB-R7943 significantly reduced Ca^2+^ entry response in TRPC6-expressing, but not in TRPC6-knocked down MCs. Ca^2+^ entry response was also significantly attenuated in Na^+^ free solution. Single knockdown of TRPC6 and TRPC1 resulted in a comparable suppression on Ca^2+^ entry with double knockdown of both. These results suggest that TRPC6 may regulate GFR by modulating MC contractile function through multiple Ca^2+^ signaling pathways.

## Introduction

Glomerular filtration is controlled by intra- and extra-glomerular factors. Among the intra-glomerular factors, the tone of mesangial cells (MCs) plays a role in regulating glomerular filtration rate (GFR)^1, 2^. MCs constitute the central stalk of the glomerulus and are attached to glomerular basement membrane by anchoring filaments. Like vascular smooth muscle cells, MCs possess a contractile phenotype which enables them to alter intraglomerular capillary blood flow and the glomerular ultrafiltration surface area, and thereby GFR^3^. Impairment of the MC contractile function may contribute to hyperfiltration in early diabetes mellitus^4–6^.

Canonical transient receptor potential (TRPC) 6 belongs to the seven-member family of TRPCs and functions as a Ca^2+^-conductive cation channel^7^. It is well documented that TRPC6 in vascular smooth muscle cells regulates vascular tone by modulating intracellular Ca^2+^ signaling^8–12^. The importance of TRPC6 in renal physiology and pathophysiology has been recently acknowledged. TRPC6 is a critical component of the slit diaphragm which is a major barrier for plasma protein filtration in glomeruli^13^. Several “gain-of-function” mutations of TRPC6 in glomerular podocytes are the cause of familial focal segmental glomerulosclerosis^13–17^. Over expression of TRPC6 in podocytes is associated with acquired forms of proteinuric kidney disease^18^. In our previous studies, we demonstrated that TRPC6 was expressed in MCs^19^ and participated in Ang II-stimulated Ca^2+^ response and contraction of MCs^4, 5^. However, whether the TRPC6-dependent cellular responses of MCs can be translated into a mechanism for regulation of GFR is unknown. The aim of the present study was to determine if removal of TRPC6 in kidneys/MCs could alter GFR by attenuating Ca^2+^ signaling in MCs. Our results from TRPC6 knockout mice and kidney TRPC6-knocked down rats support an indispensable role of TRPC6 in regulating glomerular filtration function.

## Results

### GFR is elevated in TRPC6 knockout mice

**Figure 1:**
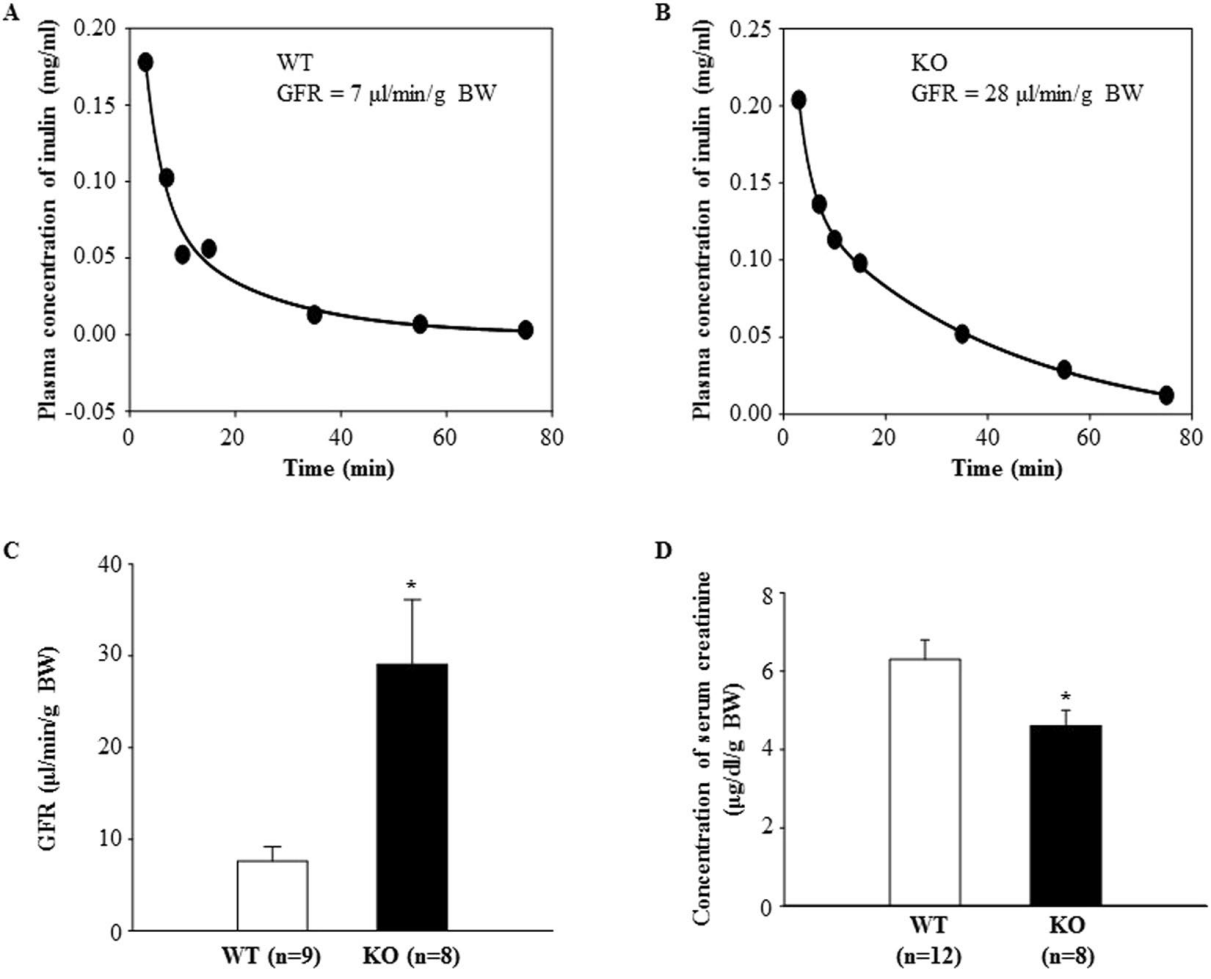
GFR measurements in conscious TRPC6 WT and KO mice. (**A** and **B**): representative plasma clearance kinetics of FITC-inulin in a WT (**A**) and KO (**B**) mouse. (**C**) Summarized GFR in WT and KO mice. (**D**) Concentration of **s**erum creatinine in WT and KO mice.

A two-compartment clearance model was used to estimate GFR in conscious TRPC6 knockout (KO) and wild type (WT) control mice. At resting state, the decay rate of the plasma inulin was remarkably faster in KO mice compared to WT mice (Fig. 1A & B). The calculated GFR in KO mice was significantly greater than that in WT mice (29.1 ± 7.1 μl/min/g BW vs. 7.6 ± 1.6 μl/min/g BW, KO vs. WT, P < 0.01) (Fig. 1C). Consistent with this, the level of serum creatinine in KO mice was significantly lower than that in WT mice (Fig. 1D).

### TRPC6 knockout did not change arterial blood pressure and urinary albumin excretion

**Figure 2:**
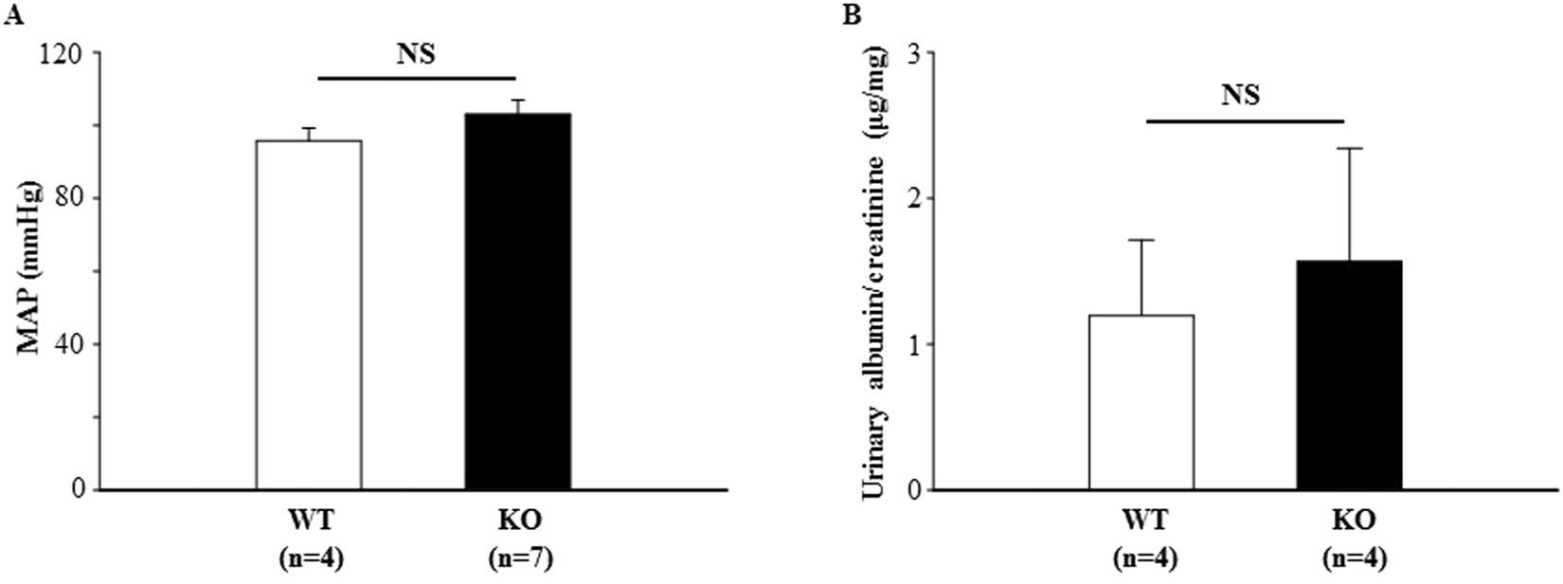
Influence of TRPC6 knockout on arterial blood pressure and urinary albumin excretion. (**A**) Mean arterial blood pressure (MAP) in WT and KO mice measured by radiotelemetry. (**B**) Urinary albumin excretion in WT and TRPC6 KO mice. NS indicates no significant difference, KO vs. WT. “n” indicates the number of mice in each group.

It has been reported that TRPC6 KO mice are hypertensive due to a compensatory upregulation of TRPC3 in vasculatures^20^. In the present study, we measured baseline arterial blood pressure by telemetry. In contrast to the findings by Dietrich *et al*.^20^, but consistent with Eckel *et al*.^21^, there was no difference in mean arterial pressure (MAP) between KO and WT mice (Fig. 2A). Similarly, urinary albumin excretion rate in KO mice was comparable to that in WT mice (Fig. 2B).

**Figure 3:**
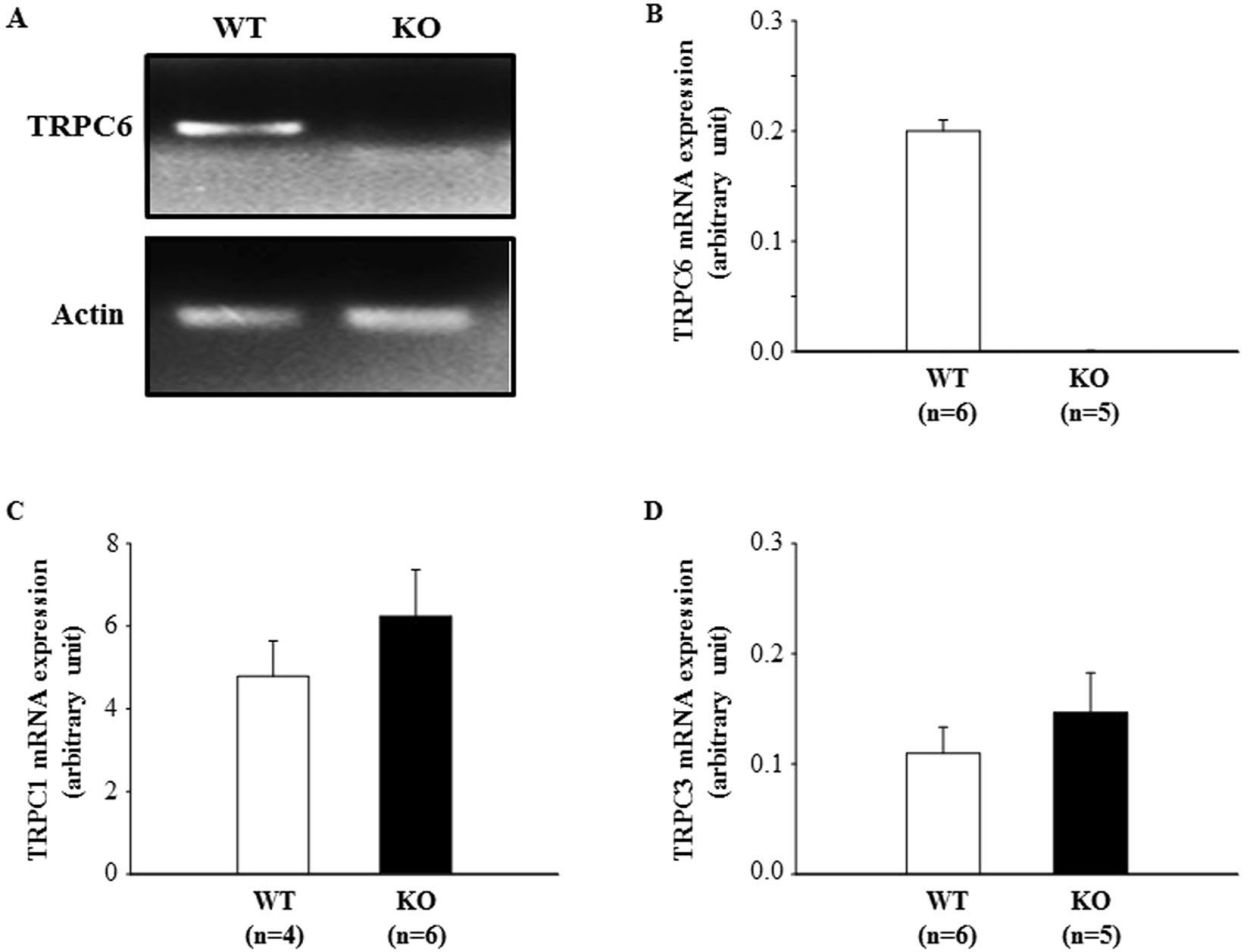
mRNA expression of TRPC1, TRPC3, and TRPC6 in the renal cortex of WT and KO mice. (**A**) A representative image or TRPC6 mRNA bands. An original and full-length gel showing TRPC1, TRPC3 and TRPC6 mRNA bands is presented in Fig. 1S of Supplementary Information. (**B–D**) Summary data of quantitative real time RT-PCR. Actin was used as a control.

RT-PCR and quantitative real-time RT-PCR using renal cortex extracts showed that TRPC6 mRNA was undetectable in KO mice. There was no statistically significant difference in mRNA expression levels of TRPC1 and TRPC3, the other two native TRPC isoforms in MCs^19^, between KO and WT mice (Fig. 3).

### Ang II-induced GFR decline was attenuated in rats whose renal TRPC6 was knocked down

**Figure 4:**
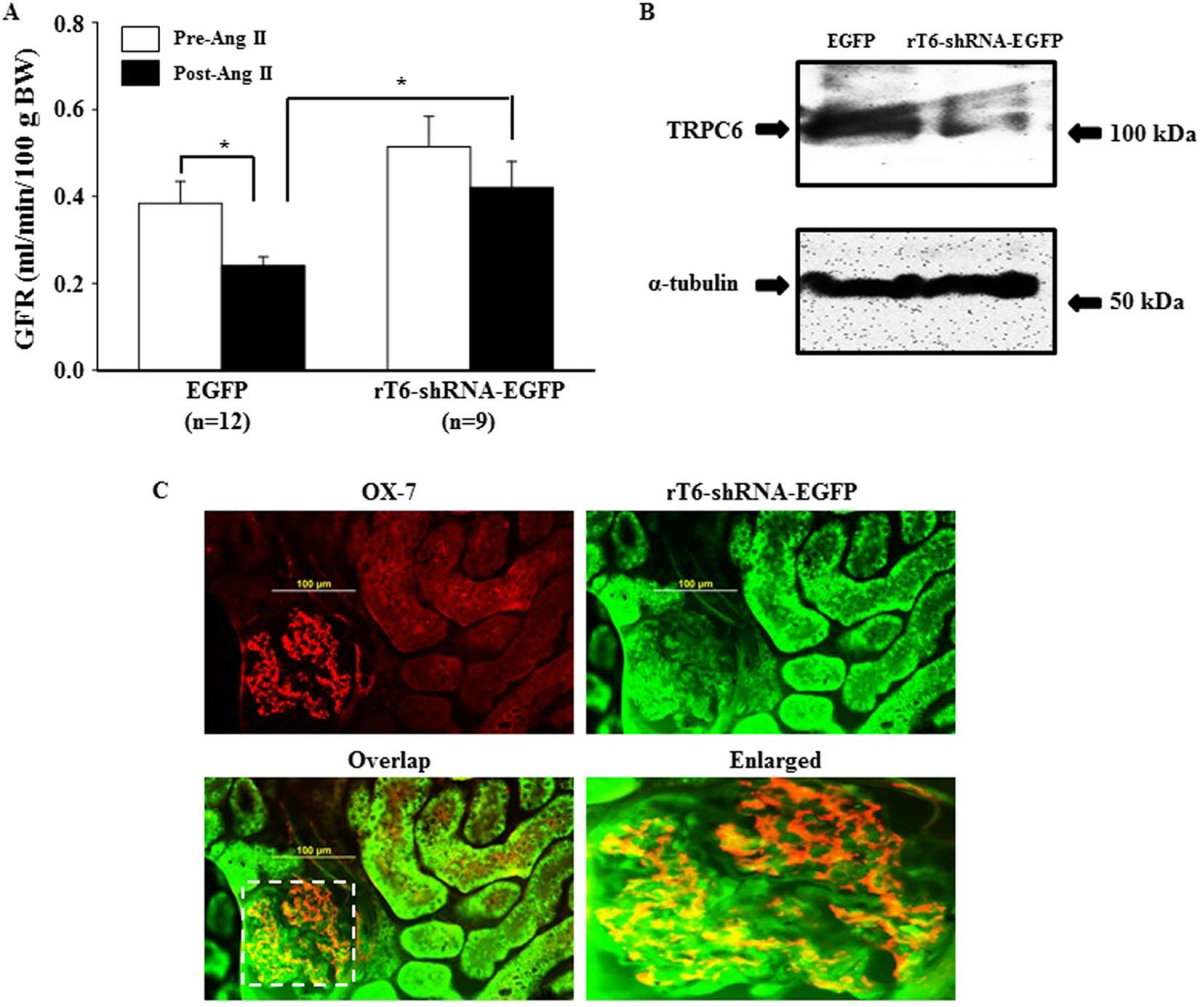
Effect of *in vivo* knockdown of TRPC6 in kidney on Ang II-induced GFR response in rats. Rats were delivered with EGFP plasmid (Control) or EGFP-tagged shRNA construct against rat TRPC6 (rT6-shRNA-EGFP) into the left kidney. GFR was measured 4 days after treatment. (**A**) GFR in rats treated with EGFP alone and rT6-shRNA-EGFP before (Pre-Ang II) and after (Post-Ang II) Ang II infusion. *Denotes P < 0.05, comparison between the groups as indicated. (**B**) Representative Western blot, showing TRPC6 protein expression in the renal cortical tissues of the left kidney (transfected kidney) from EGFP alone or rT6-shRNA-EGFP treated rats. α-tubulin was used as a loading control. (**C**) Immunofluorescence staining, showing distribution of rT6-shRNA-EGFP (green) in kidney. Glomerular MCs were labeled with OX-7, shown by red signals. Positively transfected MCs are indicated by yellow fluorescence in the panels of Overlap. The right bottom panel is an enlarged image of the region indicated by a rectangle in the panel of Overlap.

To further determine a role of TRPC6 in kidney in regulation of GFR, we delivered the EGFP-tagged shRNA constructs specifically against rat TRPC6 (rT6-shRNA-EGFP) into the left kidney via the left renal artery. The control rats were delivered with EGFP plasmid alone through the same route. Similar to TRPC6 KO mice, the baseline GFR in rT6-shRNA-EGFP treated rats was higher than that in EGFP-treated rats although the difference is not statistically significant (Fig. 4A). Importantly, the GFR responses to Ang II stimulation were significantly different between the two groups. Continuous infusion of Ang II (1.7 ng/min/100 g BW) for 1 h significantly reduced GFR in rats treated with EGFP plasmid alone. However, Ang II failed to significantly decrease GFR in the rats with local knockdown of TRPC6. Moreover, the GFR after Ang II treatment was also significantly greater in the renal TRPC6-knocked down than in the control rats (Fig. 4A). Western blot verified that TRPC6 protein abundance was markedly reduced in the rT6-shRNA-EGFP treated kidney compared to control kidney (Fig. 4A). Immunofluorescence staining showed that the rT6-shRNA constructs were distributed to both glomerulus and tubules. However, consistent with a previous study using a similar plasmid delivery approach^22^, a large population of MCs inside glomerulus were positively transfected with the rT6-shRNA constructs (Fig. 4C). These results are consistent with the data from mice and suggest that TRPC6 could regulate GFR.

### Contractile function of TRPC6 deficient MCs is impaired

**Figure 5:**
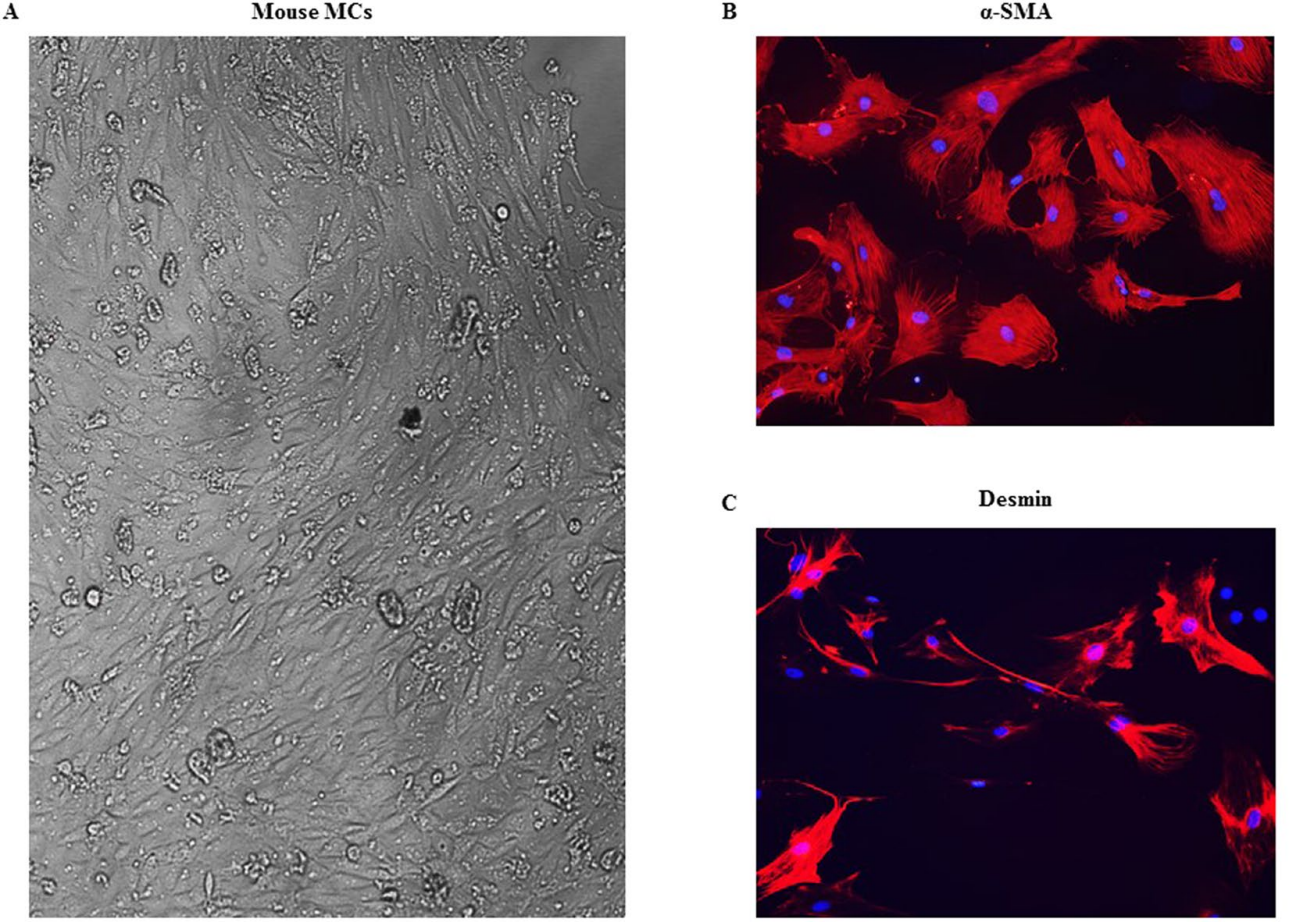
Identification of mouse MCs. (**A**) Monolayer of spindle shaped MCs. (**B**) Staining MCs for α-smooth muscle actin (red). (**C**) Staining MCs for desmin (red). In both B and C, cell nuclei were stained with DAPI (blue). Magnification: × 100.

**Figure 6:**
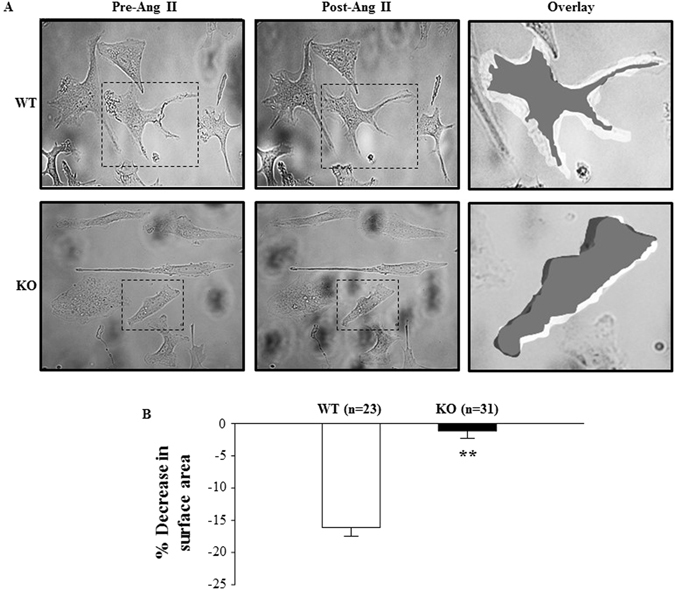
Ang II-induced contraction of primary mouse MCs isolated from TRPC6 WT and KO mice. (**A**) Representative morphology of MCs before and 30 min after 1 μM Ang II stimulation. The changes in cell size by Ang II are illustrated on the right panel (Overlay), by overlapping the images of the same cell before and after Ang II treatment using Photoshop software. The cells before Ang II treatment were white and after treatment black. The right panels are enlarged regions indicated by the dashed rectangles in the left and middle panels. (**B**) Summary data in 23 normal MCs from 8 WT mice and 31 TRPC6 deficient MCs from 10 KO mice, showing the Ang II-induced decreases in the surface area of MC with and without TRPC6. **Denotes P < 0.01, KO vs. WT.

We have previously demonstrated that alterations of MC contraction could change GFR^1, 23^. Figure 4C showed that a large population of MCs were positively transfected with TRPC6 shRNA, which resulted in an increase in GFR both under basal condition and after Ang II treatment. To determine if TRPC6 plays a role in MC contraction, we examined Ang II-stimulated contraction of MCs isolated from WT and TRPC6 KO mice. Primary mouse MCs were identified by their appearance as elongated and spindle shaped cells growing in parallel arrays, their abundant parallel α-smooth muscle actin throughout the cytoplasm, and their positive staining with desmin as described in^24, 25^ (Fig. 5). As shown in Fig. 6, the Ang II-induced contractile response was significantly lower in TRPC6 deficient cells. These results are consistent with our previous report that the contraction of human MCs was enhanced by overexpression of TRPC6, but suppressed by knockdown of TRPC6^5^, and suggest a significant contribution of TRPC6 to MC contractile function.

### TRPC6 mediated vasoconstrictor-induced Ca^2+^ entry in MCs

**Figure 7:**
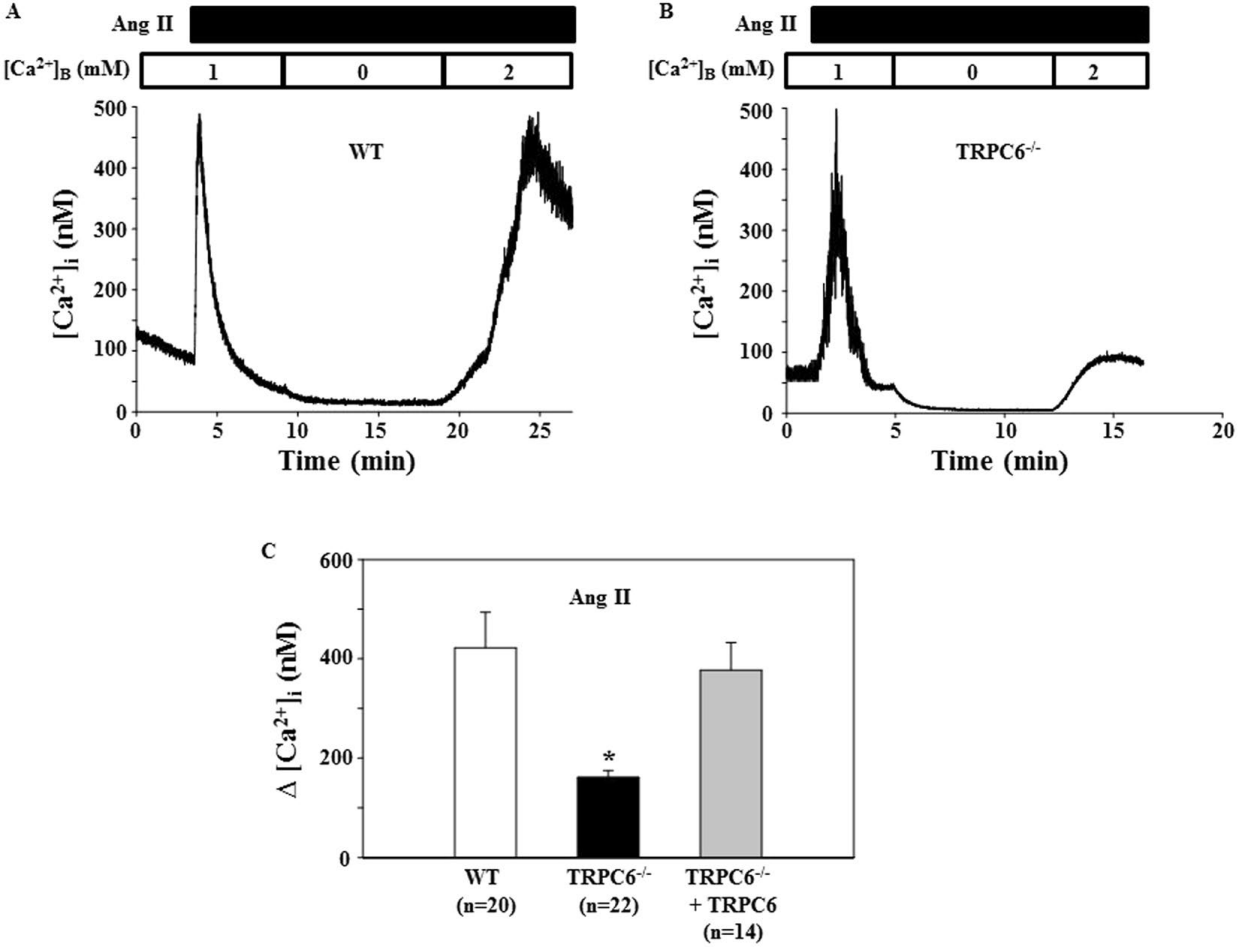
Ang II-induced Ca^2+^ response in primary mouse MCs. (**A** and **B**) Representative traces, showing [Ca^2+^]_i_ in response to 1 μM Ang II in the MC isolated from a WT (**A**) and TRPC6^−/−^ (**B**) mouse. [Ca^2+^]_B_ indicates the Ca^2+^ concentration in bathing solution. Application of Ang II is indicated by a horizontal bar at the top. (**C**) Summarized Ca^2+^ entry response to 1 μM Ang II in WT, TRPC6^−/−^ mouse MCs, and TRPC6^−/−^ mouse MCs introduced with rat *trpc6*. Δ[Ca^2+^]_i_ was calculated by subtracting [Ca^2+^]_i_ before re-addition of 2 mM Ca^2+^ to the bath from the peak [Ca^2+^]_i_ after re-addition. The numbers inside the parentheses represent the number of cells analyzed from 6 WT and 6 KO mice. *P < 0.05, compared to both WT and TRPC6^−/−^ + TRPC6.

**Figure 8:**
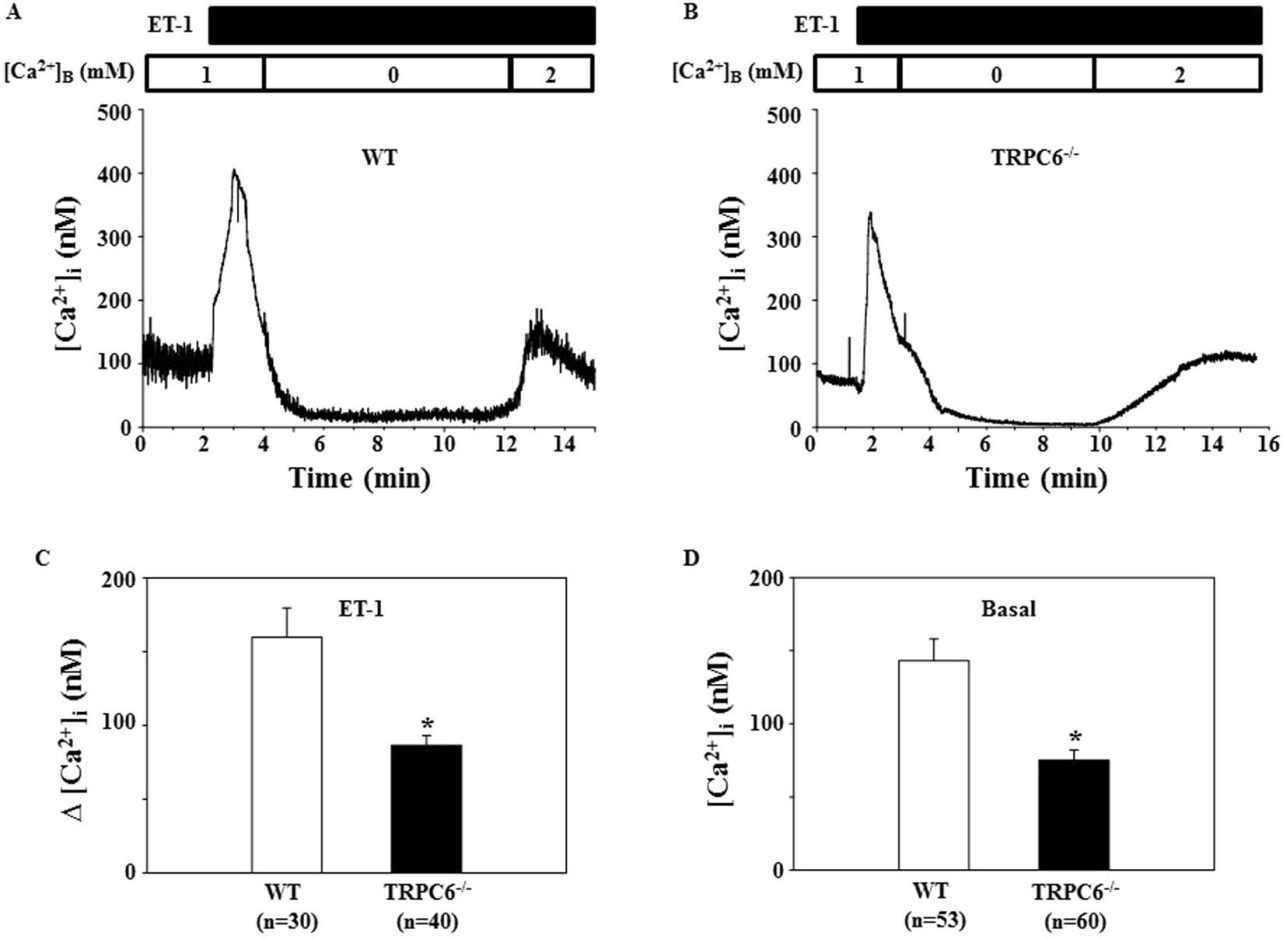
Endothelin-1-induced Ca^2+^ response in primary mouse MCs. (**A** and **B**) Representative traces, showing [Ca^2+^]_i_ in response to 100 nM endothelin-1 (ET-1) in the MC isolated from a WT (**A**) and TRPC6^−/−^ (**B**) mouse. [Ca^2+^]_B_ indicates the Ca^2+^ concentration in bathing solution. Application of ET-1 is indicated by a horizontal bar at the top. (**C**) Summarized Ca^2+^ entry response from experiments presented in A and B. Δ[Ca^2+^]_i_ was calculated by subtracting [Ca^2+^]_i_ before re-addition of 2 mM Ca^2+^ to the bath from the peak [Ca^2+^]_i_ after re-addition. The numbers inside the parentheses represent the number of cells analyzed from 6 WT and 6 KO mice. *P < 0.05, compared to both WT. (**D**) The basal [Ca^2+^]_i_ (before administration of Ang II in Fig. 6 and ET-1 in Fig. 7A–C) in TRPC6−/− and WT mouse MCs. “n” indicates the number of cells analyzed in each group.

Like vascular smooth muscle cells, MC contraction is triggered by an elevation of [Ca^2+^]_i_. We postulated that TRPC6 contributes to MC contraction by mediating Ca^2+^ entry. To test this hypothesis, we assessed [Ca^2+^]_i_ changes in response to Ang II in fura-2 loaded primary MCs isolated from WT and TRPC6 KO mice. Consistent with previous studies^1, 23^, Ang II evoked a rapid and transient increase in cytosolic Ca^2+^ in the presence of extracellular Ca^2+^. Removal of extracellular Ca^2+^ immediately reduced the [Ca^2+^]_i_ to a level lower than baseline (Fig. 7A & B). Re-addition of Ca^2+^ to the bath led to Ca^2+^ entry, resulting in an elevation of [Ca^2+^]_i_. The profiles of Ang II response were similar in both WT and KO MCs. However, the Ca^2+^ entry response upon re-addition of Ca^2+^ was significantly lower in the TRPC6 deficient MCs (Fig. 7A–C). Furthermore, the attenuated Ca^2+^ entry response was rescued by re-introducing *trpc6* to the *trpc6* deleted cells (Fig. 7C). Similarly, the Ca^2+^ entry response to endothelin-1, another vasoconstrictor was also significantly lower in the TRPC6^−/−^ MCs compared to WT cells (Fig. 8A–C). We also found that the basal level of intracellular Ca^2+^ in TRPC6 deficient MCs was significantly lower than that in WT mouse MCs (Fig. 8D). These results suggest that TRPC6 contributed to the basal intracellular Ca^2+^ concentration and participated in vasoconstrictor-stimulated Ca^2+^ influx in MCs.

### Na^+^/Ca^2+^ exchanger (NCX) and other TRPC proteins were involved in TRPC6 dependent Ca^2+^ entry

We next studied the molecular mechanism involved in TRPC6-mediated Ca^2+^ entry. These experiments were performed in commercial human MCs because these cells are easier to culture and have higher transfection efficiency compared to primary mouse MCs.

**Figure 9:**
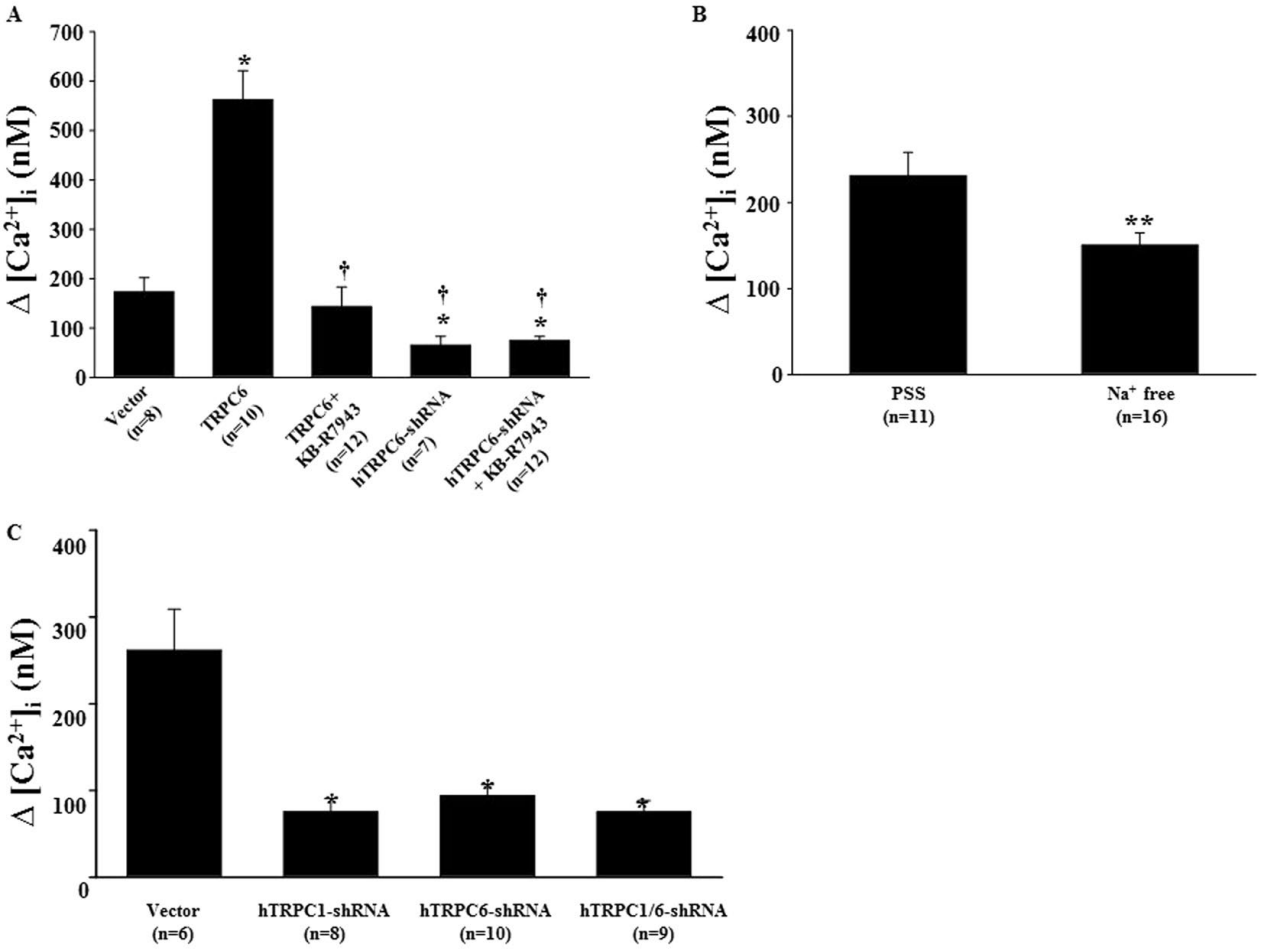
Ang II-induced Ca^2+^ response in human MCs with different treatments. (**A**) Ca^2+^ entry response (∆[Ca^2+^]_i_) in MCs transiently transfected with pSHAG (Vector) or rat TRPC6 expression plasmid (TRPC6), or shRNA construct against human TRPC6 (hTRPC6-shRNA) with and without treatment with KB-R7943 (10 μM). KB-R7943 was added to the bathing solution before re-addition of Ca^2+^. *Denotes P < 0.05, compared to Vector; †Denotes P < 0.05, compared to TRPC6. (**B**) Ca^2+^ entry response in MCs bathed in physiological saline solution (PSS) and Na^+^ free solution. **Denotes P < 0.01, PSS vs. Na^+^ free. (**C**) Ca^2+^ entry response in MCs with stable transfection of pSHAG (Vector) or shRNA construct against human TRPC1 (hTRPC1-shRNA) or hTRPC6-shRNA or both hTRPC1-shRNA and hTRPC6-shRNA. *Denotes P < 0.05, compared to Vector. In all graphs, “n” indicates the number of cells analyzed. The data shown in A to B represent at least 4 independent experiments.

It has been reported that the reverse mode of NCX contributes to TRPC6-derived Ca^2+^ response in vascular smooth muscle cells^26, 27^. To determine if the same mechanism also exists in MCs, we examined the effect of blockade of NCX on the Ang II-stimulated Ca^2+^ entry in MCs. As shown in Fig. 9A, overexpression of TRPC6 dramatically enhanced Ang II-induced Ca^2+^ influx. This response was significantly attenuated by 10 μM KB-R7943 that selectively inhibits the reverse mode NCX at this concentration^26, 27^. Knockdown of TRPC6 by transient transfection of shRNA constructs decreased the Ca^2+^ entry. With the low level of TRPC6, KB-R7943 failed to further reduce the Ca^2+^ entry (Fig. 9A). Furthermore, the Ang II-stimulated Ca^2+^ entry response was significantly reduced when the cells were bathed in a Na^+^ free solution (Fig. 9B). These results suggest that NCX may, at least partially, account for the TRPC6-mediated Ca^2+^ signaling in MCs.

We have previously demonstrated that TRPC1 mediates Ang II-stimulated Ca^2+^ entry in MCs^1^. In the present study, we found that TRPC6 also participates in the Ang II response. It is known that TRPC proteins function as a channel by forming multimeric protein complexes assembled by different TRPC isoforms^28–31^. Among 4 endogenous TRPC proteins in MCs, TRPC1 physically interacts with TRPC6^19^. Thus, TRPC1 and TRPC6 may assemble together to form a functional channel entity in MCs. If this is the case, simultaneous inhibition of both TRPC1 and TRPC6 is expected to have a similar effect on Ca^2+^ entry as the individual inhibition of either TRPC alone would have. This speculation was tested in human MCs with single knockdown of either TRPC1 or TRPC6 or double knockdown of both by stable transfection of shRNA constructs. As shown in Fig. 9C, the Ang II-stimulated Ca^2+^ influx in the empty vector-transfected cells (control) was significantly attenuated by single or double knockdown of TRPC1 and TRPC6. Importantly, the inhibition of the Ca^2+^ response was comparable among the single and double knockdown cells, suggesting that both TRPC1 and TRPC6 may be components of the same channels.

## Discussion

The importance of TRPC6 in glomerular physiology and pathology has been intensively studied by focusing on its role in protein filtration in glomeruli^13, 14, 18, 21, 32^. Overactivation of TRPC6 results in albuminuria/proteinuria by impairing podocyte structure, thereby breaking the integrity of the glomerular filtration membrane^13, 32^. In some kidney diseases, such as diabetic nephropathy, Ang II level is elevated systemically and locally. Ang II, a ligand to Gq-coupled receptor activates TRPC6 channel in podocytes^33^. Also, TRPC6 channel expression level could be upregualted by a persistently active Gq-coupled receptor signaling^34^. The enhanced TRPC6-Ca^2+^ signaling results in podocyte injury and further promotes the development of diabetic nephropathy^33^. In addition to regulation of protein permeability of the glomerular filtration barrier, the present study provided evidence that TRPC6 could also regulate water filtration in glomeruli, i.e. regulation of GFR. This conclusion is supported by a significant greater GFR and lower plasma creatinine level in TRPC6 deficient mice (Fig. 1). Thus, TRPC6 may regulate glomerular protein and water filtration differentially, i.e. increasing albumin/protein permeability but decreasing GFR.

In agreement with an earlier study on TRPC6 KO mice^21^, the present study showed no significant change in the arterial blood pressure in TRPC6 KO mice. Thus, the TRPC6 effect on GFR may be independent of its vascular effects. Instead, an intra-glomerular factor(s) may play a role. In this regard, an increase in either the permeability or the surface area of glomerular filter could raise GFR. However, TRPC6 deficiency did not affect albumin excretion rate, and did not impair the structures of podocytes, mesangial, and endothelial compartments^21^, suggesting that the GFR response in the present study may not involve a change in the glomerular filter permeability. Thus, an increase in the surface area available for filtration may contribute to the promotion of GFR by removal of TRPC6.

Glomerular MCs sit between glomerular capillary loops and maintain the structural architecture of the capillary networks^3, 35^. The contractile property of the cells confers to them a role in regulation of GFR by changing the capillary surface area available for filtration^1, 2^. Our previous studies demonstrated that TRPC6 participated in Ang II-stimulated MC contraction^5, 23^. Consistent with those findings, the Ang II-induced contraction was significantly blunted in the TRPC6-deficient MCs. We reason that the impaired TRPC6-associated MC contraction may, at least partially, contribute to the augmented glomerular filtration observed in the present study. Recently, TRPC6-mediated Ca^2+^ signal was found to promote MC apoptosis through calcineurin/NFAT and FasL/Fas signaling pathways in primary neonatal pig MCs^36^. Therefore, TRPC6 channel is multi-functional in MCs.

A role of MC TRPC6 in regulation of GFR was also supported by the data from our *in vivo* knockdown of kidney TRPC6. As described by Takabatake *et al*. and Tsujie *et al*.^22, 37^, locally delivered shRNA constructs via the renal artery were efficiently transfected into MCs. We used a similar approach to deliver shRNA constructs against rat TRPC6 into kidneys and also showed that a large portion of transfected cells were MCs. However, MCs were not the sole glomerular cells being positively transfected. Whether the other glomerular cells also contributed to the GFR response in the renal TRPC6 knocked down rats is not known. Furthermore, TRPC6 is also expressed in the tubular epithelial cells^38^ and the TRPC6 shRNA constructs in the present study were also taken up by these epithelial cells. Thus, it can not be excluded that the tubular function was altered by the knockdown of TRPC6, resulting in dysregulation of GFR by impairing the tubular-glomerular feedback mechanism.

Like vascular smooth muscle cells, MC tone is controlled by [Ca^2+^]_i_. Consistent with a role in regulation of GFR, our study supported that TRPC6 not only contributed to the basal intracellular Ca^2+^ level, but also mediated a significant portion of agonist-stimulated Ca^2+^ entry in MCs. The residual Ca^2+^ entry response in TRPC6-deficient mouse MCs (Fig. 6) and TRPC6-knocked down human MCs (Fig. 7) may be due to other native TRPC isoforms or other types of Ca^2+^ permeable channels. It is known that MCs express 4 subtypes of TRPC proteins^19^, L-type voltage-operated Ca^2+^ channels and store-operated Ca^2+^ channels^39, 40^. Different from the findings in vasculatures by Dietrich *et al*.^41^, knockout of TRPC6 did not cause compensatory upregulation of TRPC3 mRNA expression in glomeruli (Fig. 3), which is consistent with Eckel *et al*.’s findings^21^. These facts suggest that the residual response was not directly secondary to TRPC6 KO or knockdown.

Native TRPC channels are usually heteromultimers which are composed of different TRPC isoforms and the combinations of the channel complexes are tissue and cell type specific^28–31^. In MCs, the binding partner of TRPC6 is TRPC1 because the two proteins interacted and colocalized with each other in MCs^19^. Importantly, inhibition of both TRPCs had a comparable effect on Ca^2+^ response with a single inhibition of either TRPC (Fig. 7B). The present study further suggests that NCX also contributes to TRPC6-mediated Ca^2+^ entry (Fig. 7A). Thus, in MCs, there are two mechanisms underlying TRPC6-associated Ca^2+^ response. One is that Ca^2+^ itself passes through the TRPC1/TRPC6 channel complex. The other is that Na^+^ enters the cells through the TRPC1/TRPC6 channels and the resulting elevation of intracellular Na^+^ level activates NCX to extrude Na^+^ in exchange for Ca^2+^. Certainly, this conclusion does not exclude other endogenous TRPCs (TRPC3 and 4) from agonist-stimulated Ca^2+^ response through a TRPC6 independent mechanism.

With respect to the downstream mechanism of the TRPC6-mediated Ca^2+^ influx for MC contraction, the common pathway of Ca^2+^/calmodulin-myosin light chain kinase-phosphorylatin of the light chain of myosin in smooth muscle cells^42^ may apply to MCs because the two types of cells possess the same contractile phenotype^2^. In addition, the small GTPase RhoA which plays a critical role in smooth muscle contraction^42, 43^ may also contribute to the TRPC6-dependent MC contraction because RhoA is a downstream target of Ca^2+^ through TRPC6 channels^44, 45^.

In summary, the present study provides evidence that TRPC6 can regulate GFR by modulating MC tone. Considering the firm associations of podocyte TRPC6 and albumin/proteinuria^13, 14, 18, 21, 32^, the conclusion from the current study raises an interesting point that the cell type specific TRPC6 can differentially regulate glomerular function. The podocyte TRPC6 adversely affects protein permeability of the glomerular filter while the MC TRPC6 affects GFR by regulating the surface area of the filter. The cell type specific regulation of glomerular function by TRPC6 may provide a clue for intentionally manipulating the channel function in a specific cell type to treat kidney disease based on renal phenotypes.

## Methods

### Animals

All procedures were approved and performed in accordance with the guidelines and regulations of the Institutional Animal Care and Use Committee of the University of North Texas Health Science Center (UNTHSC). A total of 21 male Sprague Dawley rats and 70 male mice (35 WT and 35 KO) on C57BL/6 J background were used. All rats and mice were between 2 and 3 months of age. Rats were purchased from Harlan (Indianapolis, IN) and were randomly distributed into two groups. One group of rats (n = 12) were delivered with EGFP plasmid and the other groups of rats (n = 9) were transfected with rT6-shRNA-EGFP construct. *trpc6* ^−/−^ mice were on 129SvEv:C57Bl/6 J (50:50) crossbred background. These mice were generously provided through Charles River by Dr. Lutz Birnbaumer who originally generated the knockout mice^20^. Age-matched WT C57BL/6 mice were purchased from Charles River (Wilmington, MA). All animals were maintained at the animal facility of UNTHSC under local and National Institutes of Health guidelines.

### Measurement of GFR in conscious mice

As described in^46^ with slight modifications. Briefly, 8 *trpc6* ^−/−^ and 9 WT mice were anesthetized using isofluorane (Baxter Pharmaceutical Products, Deerfield, IL). Then, 5% FITC-inulin was injected retro-orbitally at 3.74 μl/g body weight under anesthesia within 3 seconds. After fully regaining consciousness, the mouse was restrained inside a 50-ml centrifuge tube with large air-holes drilled in the tip. Approximately 20 μl blood was collected in a heparinized capillary tube from tail veins by venipuncture using a sharp razor blade. Blood was sampled at 3, 7, 10, 15, 35, 55, 75 minutes post injection of FITC-inulin. The plasma concentration of inulin was measured using a PerkinElmer 2030 Multilabel Reader (Victor^TM^ X3, PerkinElmer Singapore Pte Ltd, Life and Analytical Sciences, Republic of Singapore) with excitation at 485 nm and emission at 538 nm and was plotted vs. time on an arithmetic scale using a nonlinear regression curve fitting program (SigmaPlot, version 11.0, Systat Software Inc., San Jose, CA). GFR was calculated based on inulin clearance using a two-compartment clearance model. The formula for GFR calculation is GFR = I/(A/α + B/β), where I is the amount (μg) of FITC-inulin delivered by the bolus injection; A and B are the Y-intercept values of the two decay rates, and α and β are the decay constants for the distribution and elimination phases, respectively.

### Measurement of arterial blood pressure in conscious mice

Blood pressure was measured in conscious mice by radiotelemetry using CA11PA-C40 transmitters (Data Science International, St. Paul, MN). Four WT and 7 KO Mice were anesthetized with ketamine (60 mg/kg) and xylazine (20 mg/kg) by intraperiteal injection. A pressure-sensing catheter was implanted into the thoracic aorta via the left carotid artery. The transducer unit was implanted in a subcutaneous pouch along the right flank. Blood pressure was measured 7 days after surgery. During blood pressure measurements, the mice were housed in a quiet monitoring room in the animal facility. Data were sampled at 10 seconds/10 min for 24 hours, and analyzed using the software provided by the vendor.

### Serum creatinine assay

Serum creatinine level was measured in O’Brien Kidney Center at University of Texas Southwestern Medical Center by P/ACE MDQ Capillary Electrophoresis System (Beckman Coulter, Inc., Fullerton, CA). Mice were transiently anesthetized with isofluorane and blood samples (~100 μl) were collected from retro-orbital venous plexus. Blood was set aside for 45 min at room temperature for clotting and then, was centrifuged to separate the serum. The samples were stored at −80 °C freezer until assay was conducted.

### Urinary albumin excretion assay

Urine samples were collected from the bladder of wild type (WT) and TRPC6 knockout (KO) mice. Urine albumin levels were measured using commercial ELISA kit (Exocell Inc., Philadelphia, PA) and were normalized to urinary creatinine (Creatinine companion kit, Exocell Inc., Philadelphia, PA) following instructions provided by manufacturer.

### *In vivo* delivery of constructs into the kidney of rats

We employed the approach described by Takabatake *et al*. and Tsujie *et al*. with modification^22, 37^. In brief, rats were anesthetized with pentobarbital (50 mg/kg, i.p.). The left kidney and renal artery were surgically exposed with a mid-line incision, and a 24-gauge catheter was inserted into the renal artery. After the proximal site of the abdominal aorta was clamped, the left kidney was perfused with balanced salt solution (BSS) followed by injection of 0.5 ml BSS containing either EGFP or rT6-shRNA-EGFP constructs at 0.4 μg/g body weight. The renal vein was clamped immediately after injection. About 10 min later, the catheter was removed and the puncture was pressed to stop bleeding. The terminal experiments were carried out 4 days after surgery.

### Measurement of GFR in rats

As described in our previous studies^1, 23^. In brief, all rats were anesthetized by intraperitoneal injection of pentobarbital (50 mg/kg). The left jugular vein was cannulated with a PE-10 tubing for infusion of fluid and chemicals. The right carotid artery was cannulated with a PE-10 tubing for collecting blood samples. Urine was collected through a PE-50 tubing implanted in the bladder. In all rats, physiological saline solution (PSS) containing 10 mg/ml FITC-inulin was infused at a rate of 1 ml/h/100 g body weight. After a 1-hour equilibration period, a blood sample (~100 μl) was taken and urine was collected during the next 30-min period. Then, the inulin-PSS solution containing 1 μM Ang II was infused into the rats (1.7 ng/min/100 g body weight). After a 30 min equilibration period, a 30-min urine sample was collected again. At the end of the period, a larger plasma sample was taken. Urinary volume was determined gravimetrically. GFR was calculated on the basis of urinary volume, urine and plasma inulin concentrations. Concentrations of inulin was measured using the PerkinElmer 2030 Multilabel Reader (Victor^TM^ X3, PerkinElmer Singapore Pte Ltd, Life and Analytical Sciences, Republic of Singapore) with excitation at 485 nm and emission at 538 nm.

### Mouse MC isolation

Ten WT and 12 KO mice were used for isolation of MCs. Which were used for contraction assays and Ca^2+^ imaging experiments. Mice were euthanized with intraperitoneal injection of ketamine (60 mg/kg) and xylazine (20 mg/kg), and kidneys were removed immediately for isolation of glomeruli. The glomerulus pellet was resuspended with 2 ml Hank’s solution containing 1500 unit of collagenase IV (Sigma, St. Louise, MO, catalog number: C-1889) and incubated at 37 °C water bath for 5 min gently agitating periodically. After the 5 min incubation, 3 ml Hank’s solution was added to make a total volume of 5 ml cell suspension. The cell suspension was then centrifuged and resuspended with 5 ml DMEM medium for 3 times, and then transferred to a 25 cm^2^ flask and added 20 μl BM-cyclin 1 and placed in a cell culture incubator. After 3 days, the media in the flask was collected, centrifuged, and resuspended with fresh DMEM media. The new cell suspension was transferred back to the flask with addition of 20 μl BM-cyclin 2. This process was repeated 3 times every 4 days with alternate addition of 20 μl BM-cyclin 1 and 2, but no BM-cyclin in the last time. Cells of subpassage 3 to 5 were used in the present study.

### Cell culture and transfection

Human MCs were purchased from Lonza (Walkersville, MD). Both human and primary mouse MCs were cultured in low glucose (5.6 mM) DMEM media (Gibco, Carlsbad, CA) supplemented with 25 mM HEPES, 4 mM glutamine, 1.0 mM sodium pyruvate, 0.1 mM nonessential amino acids, 100 U/ml penicillin, 100 µg/ml streptomycin and 20% FBS. For human MCs, only subpassages less than eleven generations were used in the present study while for the primary mouse MCs, sub-passage 3 to 5 were used.

For transient transfection, all plasmids were transiently transfected into human MCs using LipofectAmine and Plus reagent (Invitrogen-BRL, Carlsbad, CA) following the protocols provided by the manufacturer. Cells were used for functional experiments 72 hours after transfection. For stable transfection, the TRPC1 and TRPC6 shRNA constructs were transfected into human MCs individually or simultaneously using the approach described above. Since these constructs contain kanamyocin and/or hygromycin selectable markers, the antibiotics were added to cell culture medium 48 hours after transfection. Cells surviving after at least 1 week were used for functional experiments.

### MC contractility assay

Ang II-induced MC contraction was measured by changes in planar surface area as described in our previous publication^1, 23^. Percent decrease in surface area was calculated as [(the surface area of MC after Ang II − the surface area of the cell before Ang II)/the surface area before Ang II] × 100%.

### Fluorescence measurement of [Ca^2+^]_i_

Measurements of [Ca^2+^]_i_ in MCs using fura-2 were performed using dual excitation wavelength fluorescence microscopy as described in^23^.

### Quantitative real time RT-PCR

The total RNA was isolated from mouse kidney cortex using High Pure RNA Tissue Kit (Roche, Germany) following the manufacturer’s protocol. Primers used in the present study were: 1) TRPC1: forward: CACTCGTTCATTGGCACCTGCTTT and reverse: GCAGCTTCGTCAGCACAATCACAA; 2) TRPC3: forward: TGCACACTGCCATAAGTACGAGGT and reverse: GTCAAGCCTTTG TTTCTCAGCGCA; 3) TRPC6: forward: ACTGTGTGGATTACATGGGCCAGA and reverse: AGGATTGCCTCCACAATCCGTACA; 4) actin: forward: TGTGATGGTGGGAATGGGTCAGAA and reverse: TGTGGTGCCAGATCTTCTCCATGT. All primers were designed and synthesized by IDT (Coralville, Iowa). RT reactions used iScript cDNA synthesis kit (BioRad, Hercules, CA) with 1.0 μg total RNA in a final volume of 20 μl following the manufacturer’s protocol. Real time PCR used 0.2 μg RT product, 100 nM primers, and was performed using iQ SYBR green supermix (BioRad, Hercules, CA) in a final volume of 20 μl. The PCR mix was denatured at 95 °C for 15 min, followed by 45cycles of melting at 94 °C for 12 s, annealing at 50 °C for 30 s and elongation at 72 °C for 30 s and 80 °C for 10 s. After amplification, a melting curve analysis from 65 °C to 95 °C with a heating rate of 0.5 °C/s with a continuous fluorescence acquisition was made. The assay was run on a C1000^TM^ Thermal Cycler (BioRad, Hercules, CA). The average C_t_ (threshold cycle) of fluorescence unit was used to analyze the mRNA levels. The mRNA levels of TRPC1, TRPC3, and TRPC6 were normalized by actin mRNA levels. Quantification was calculated as follows: TRPC mRNA level = 100 × 2^ΔCT^, where ΔCT = C_T, actin_ − C_T, TRPC_.

### Fluorescence immunocytochemistry

Mouse MCs were fixed with 4% paraformaldehyde and incubated with either α-smooth muscle actin or desmin mouse monoclonal primary antibody at 1:50 at 4 °C overnight. The cells were then incubated with secondary antibodies (goat anti-mouse conjugated with Alexa Fluor 568) at a concentration of 1:500 for 1 h at room temperature. ProLong Gold anti-fade reagent with DAPI was used as a nuclear stain and mount reagent. Staining was visualized and images were captured by confocal laser-scanning microscope (Zeiss LSM410).

### Fluorescence immunohistochemistry

As described in our previous publication^47^. In brief, the paraformaldehyde-fixed kidney was sectioned at 4-μm thickness (Cryostat 2800 Frigocut-E, Leica Instruments). Anti-CD90/thymic antigen 1 mouse monoclonal antibody (MRC OX-7) at 1:100 and Alexa Fluor 568 goat anti-mouse IgG (Invitrogen, Grand Island, NY) at 1:2000 were used to label glomerular MCs. Staining was visualized using a confocal laser-scanning microscope (Zeiss LSM510).

### Materials

KB-R7943 was purchased from Calbiochem (La Jolla, CA). All other chemicals were purchased from Sigma-Aldrich (Sigma, ST. Louis, MO) unless indicated in other places. Anti-α-smooth muscle actin (catalog no: A5228) and anti-desmin (catalog no: D8281) antibodies were purchased from Sigma-Aldrich (Sigma, ST. Louis, MO). Anti-CD90/thymic antigen 1 mouse monoclonal antibody (MRC OX-7) was purchased from Abcam Inc. (Cambridge, MA). The shRNA constructs against human TRPC1 (hTRPC1-shRNA) was obtained from Dr. Leonidas Tsiokas at University of Oklahoma. The shRNA constructs against human TRPC6 (hTRPC6-shRNA) were a kind gift from Dr. M. J. Villereal at the University of Chicago^48^. The rat TRPC6 expression plasmids were obtained from Dr. David Saffen at Ohio State University^49, 50^.

### Statistical Analysis

Data were reported as means ± SE. The one-way ANOVA plus Student-Newman-Keuls post-hoc analysis, Student unpaired t-test, and Student paired t-test were used to analyze the differences among multiple groups, between two groups, and before and after treatment in the same group, respectively. P < 0.05 was considered statistically significant.

## Electronic supplementary material

Supplementary Information
