# Supplementary material for: Increased glomerular filtration rate and impaired contractile function of mesangial cells in TRPC6 knockout mice
Source: Sci Rep. 2017 Jun 23;7:4145. doi: 10.1038/s41598-017-04067-z (PMC5482875; doi:10.1038/s41598-017-04067-z)
Supplement: Supplementary file 1 — Supplementary Information [file 41598_2017_4067_MOESM1_ESM.pdf]

Supplementary Information

INCREASED GLOMERULAR FILTRATION RATE AND IMPAIRED CONTRACTILE FUNCTION OF MESANGIAL CELLS IN TRPC6 KNOCKOUT MICE

Weizu Li[2], Yanfeng Ding[1], Crystal Smedley[1], Yanxia Wang[1], Sarika Chaudhari[1],

Lutz Birnbaumer[3] and Rong Ma[1*]

Fig. 1S

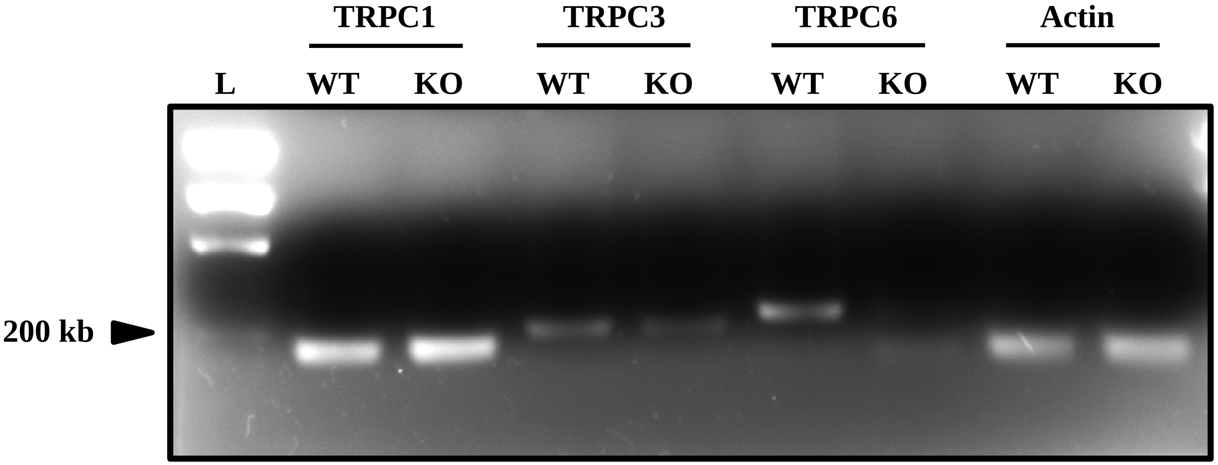

FIGURE LEGENDS

Fig. 1S A representative original and full-length gel, showing mRNA bands of TRPC1, TRPC3, and TRPC6 in the renal cortex of WT and KO mice. Actin was used as a control.
